# Outbreak of autochthonous cases of malaria in coastal regions of Northeast Brazil: the diversity and spatial distribution of species of *Anopheles*

**DOI:** 10.1186/s13071-020-04502-7

**Published:** 2020-12-14

**Authors:** Elainne Christine de Souza Gomes, Derciliano Lopes da Cruz, Maria Alice Varjal Melo Santos, Renata Maria Costa Souza, Cláudia Maria Fontes de Oliveira, Constância Flávia Junqueira Ayres, Renata Martins Domingos, Maria das Graça da Silva Pedro, Marcelo Henrique Santos Paiva, Lílian Maria Lapa Montenegro Pimentel

**Affiliations:** 1 Department of Parasitology, Aggeu Magalhães Institute, Fiocruz, Ministry of Health, Brazil, Av. Professor Moraes Rego, s/n Cidade Universitária, Recife, PE 50740-465 Brazil; 2 Department of Entomology, Aggeu Magalhães Institute, Fiocruz, Ministry of Health Brazil, Av. Professor Moraes Rego, s/n Cidade Universitária, Recife, PE 50740-465 Brazil; 3 Department of Immunology, Aggeu Magalhães Institute, Fiocruz, Ministry of Health, Brazil, Av. Professor Moraes Rego, s/n Cidade Universitária, Recife, PE 50740-465 Brazil; 4 Health Department of Conde/ Paraíba, Rua Paulo da Rocha Barreto, 79 Centro, Conde, PB 58322-000 Brazil; 5 Agreste Academic Center, University of Pernambuco, Rodovia BR-104, km 59 Nova Caruaru, Caruaru, PE 55002-970 Brazil

**Keywords:** Malaria, *Anopheles*, Autochthonous transmission, Outbreak, Extra-amazon, *Plasmodium vivax*, Spatial analysis

## Abstract

**Background:**

Brazil has the fourth highest prevalence of malaria of all countries in the Americas, with an estimated 42 million people at risk of contracting this disease. Although most cases occur in the Amazon region, cases of an autochthonous nature have also been registered in the extra-Amazonian region where *Anopheles aquasalis* and *An. albitarsis* are the mosquito species of greatest epidemiological interest. In 2019, the municipality of Conde (state of Paraíba) experienced an epidemic of autochthonous cases of malaria. Here we present preliminary results of an entomological and case epidemiology investigation, in an attempt to correlate the diversity and spatial distribution of species of *Anopheles* with the autochthonous cases of this outbreak of malaria.

**Methods:**

Case data were collected using case report forms made available by the Conde Municipal Health Department. The entomological survey was carried out from July to November 2019. The various methods of capture included the use of battery-powered aspirators, mouth aspirators, Shannon traps, BG-Sentinel traps (with and without dry ice) and CDC light traps. Captured mosquitoes were separated, packaged and sent to the laboratory for sexing and molecular identification of the various species of anophelines. The data were tabulated and analyzed using Microsoft Excel. Spatial analysis of the data was performed using ArcGis 10 software.

**Results:**

In 2019, 20 autochthonous cases and one imported case of malaria caused by *Plasmodium vivax* were diagnosed, with three cases of relapses. A total of 3713 mosquitoes were collected, of which 3390 were culicines and 323 were anophelines. Nine species of genus* Anopheles* were identified, with the most abundant being *An. aquasalis* (38.9%), followed by *An. minor* (18.2%) and *An. albitarsis* (9.0%). Spatial analysis of the data showed that the area could be considered to be at risk of malaria cases and that there was a high prevalence of *Anopheles*.

**Conclusions:**

The results presented indicate that this extra-Amazonian region has an environment conducive to maintenance of the malaria transmission cycle owing to the wide diversity of *Anopheles* species. This environment in combination with the high influx of people from endemic areas to the study area provides a perfect setting for the occurrence and maintenance of malaria. 
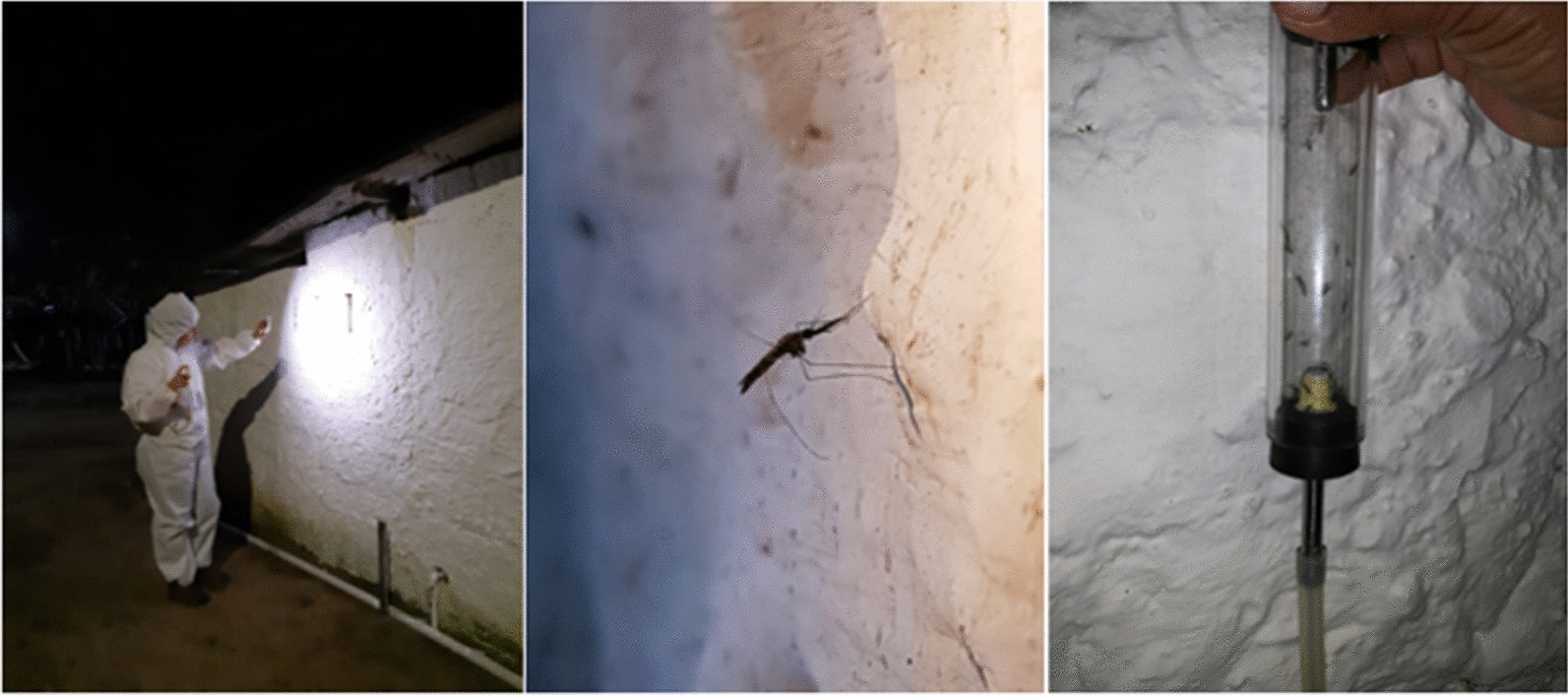

## Background

Malaria is the most prevalent infectious disease caused by parasites in the world. According to the World Health Organization, in 2018, 228 million cases occurred worldwide, 93% of them in Africa [1]. Most cases are caused by infective *Plasmodium falciparum*, but in the Americas, *Plasmodium vivax* is responsible for the transmission of 75% of all malaria cases [1]. On the South American continent, most cases occur in the Amazon region, and Brazil ranks fourth in terms of malaria prevalence, only behind Venezuela, Peru and Bolivia [2]. It is estimated that about 42 million people in Brazil are at risk of contracting malaria, and in 2018 more than 200,000 cases were registered [1], with 99.7% of these concentrated in the Amazon region [3]. From a territorial perspective, malaria divides Brazil into two parts: the endemic area, including all states in the Northern Region of the country and the states of Maranhão and Mato Grosso, and the non-endemic area (also known as the extra-Amazonian region), comprising all the other states [3], where there is residual transmission of malaria [4].

Outbreaks of malaria have been reported in the regions for years. From 2007 to 2013, four states (São Paulo [SP], Rio de Janeiro [RJ], Espírito Santo [ES] and Minas Gerais [MG]) reported 57.1% of the total of 932 autochthonous cases recorded in the extra-Amazonian region. Of this total, 34.4% were reported in ES (321 cases), 18.2% in SP (170 cases), 3.8% in RJ (35 cases) and 0.6% in MG (6 cases) [5]. From 2007 to 2016, the majority of autochthonous cases in the extra-Amazon region were also registered in the State of ES (400 cases), corresponding to 60% of the total number of cases registered in the region [6]. The State of Paraíba (PB), located in the Northeast Region of Brazil, reported 175 suspected cases of malaria from 1994 to 2018 in the Notifiable Diseases Information System (SINAN), despite not being considered an area of residual transmission. Among these, no autochthonous cases or deaths from the disease were registered [7].

Malaria is known to have a heteroxenous cycle, in which mosquitoes of the *Anopheles* genus are the biological vectors [1, 3]. In Brazil, there are about 60 species of *Anopheles* [8], of which *An. darlingi* is considered to be the main vector of human malaria [9], mostly in the Amazon region [10]. However, other species belonging to the subgenus *Nyssorhynchus* are also involved in the transmission of malaria in the Amazon, including *An. braziliensis*,* An. nuneztovari* (*s.l.*),* An. oswaldoi* (*s.l.*),* An. triannulatus* (*s.l.*) and *An. albitarsis* (*s.l.*) [6]. In the extra-Amazonian region, *An. aquasalis* and *An. albitarsis* (considered a complex of cryptic species) are of relatively greater epidemiological importance in the transmission of malaria [8].

Entomological identification of anophelines using traditional taxonomy is challenging since for many specimens it is impossible to determine the species using only the morphological characteristics of the mosquito; consequently, these species are grouped into complexes. However, the development of molecular biology techniques has facilitated species identification through the use of various types of PCR analyses [11]. Beyond simple identification, new taxonomic regroupings have even been proposed using molecular biology, such as the one proposed by Foster et al. [12] that raised the subgenera* Kerteszia*,* Lophopodomyia*,* Nyssorhynchus* and* Stethomyia* belonging to the genus *Anopheles* [13] to genus category.

However, according to the latest Brazilan State records regarding species of *Anopheles* based on classical taxonomy, 11 species of the genus *Anopheles* have been recorded in the PB, four of which are potential malaria vectors: *An. aquasalis*,* An. albitarsis*,* An. bellator* and *An. argyritarsis* [7]. The municipality of Conde, similar to many others in the coastal regions of the Northeast, contains areas of mangrovew which are a transitional coastal ecosystem between the terrestrial and marine environments. Species of *Anopheles* that reproduce in this environment exhibit a diverse pattern of behavior, feeding in peridomicile areas and resting in extradomicile ones [14].

In 2019, this municipality recorded an epidemic of autochthonous malaria, leading the Ministry of Health to launch an epidemiological and entomological investigation into the factors involved in this epidemic. In view of this, and considering the various potential vector species that inhabit the Northeast of Brazil, this study presents the preliminary results of the entomological investigation carried out in the municipality of Conde/Paraíba in an attempt to correlate the diversity and spatial distribution of Anopheles with the occurrence of these autochthonous cases. Understanding these factors are essential for explaining why malaria has returned to the Northeast and for ascertaining which risk factors may favor the maintenance of transmission in this municipality.

## Methods

### Study area

**Fig. 1:**
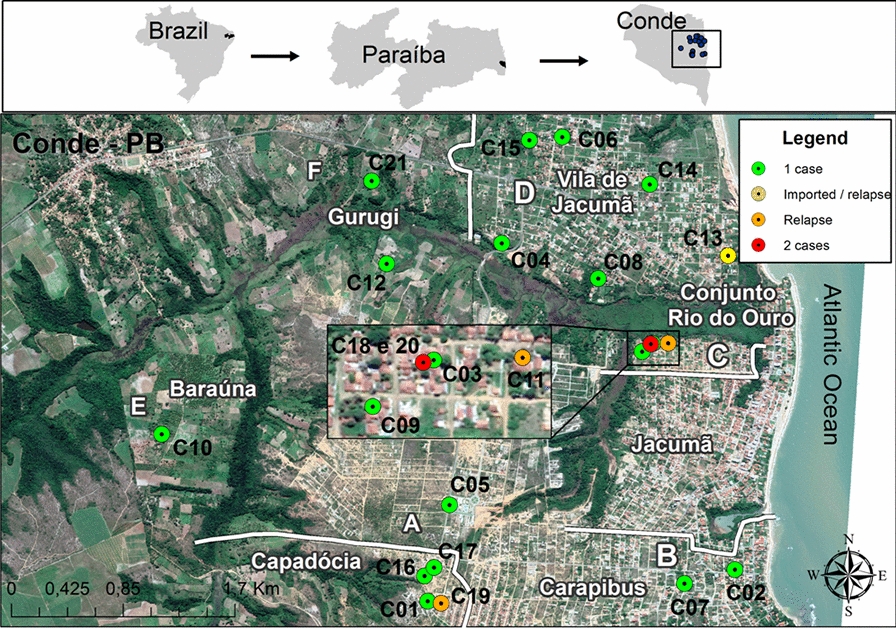
Location of the study area in Brazil, highlighting the state of Paraíba (PB) and the municipality of Conde (top). The colored circles in the inset at the top right identify the distribution of malaria cases by geolocation, with each green circle indicating one case; the yellow circle indicating one imported case from Venezuela, with relapse after treatment; the 3 orange circles indicating three cases that showed relapse after treatment; and the two red circles indicating residences where two cases were registered. The map shows the six main neighborhoods where cases occurred:* A* Capadócia,* B* Carapibus,* C* Conjunto Rio do Ouro,* D* Vila de Jacumã,* E* Baraúna,* F* Gurugi

An ecological study was carried out in the municipality of Conde, PB (Brazil). Conde is located in the Metropolitan Region of João Pessoa, along the southern coastline of the state, in the Northeast Region of Brazil (Fig. 1). It has an estimated population of 24,323, spread over approximately 173 km^2^ [15]. It is a locality covered by Atlantic Forest and is also well known for its beach tourism, welcoming many domestic and international tourists [15].

### Case data

Epidemiological case data, such as sex, age, date of case report, date of first symptoms, clinical outcome, and place of occurrence, were gathered from case report forms made available by the Municipal Health Department. All cases were also georeferenced for the purpose of creating thematic maps and spatial analysis of data.

### Mosquito collection

**Fig. 2:**
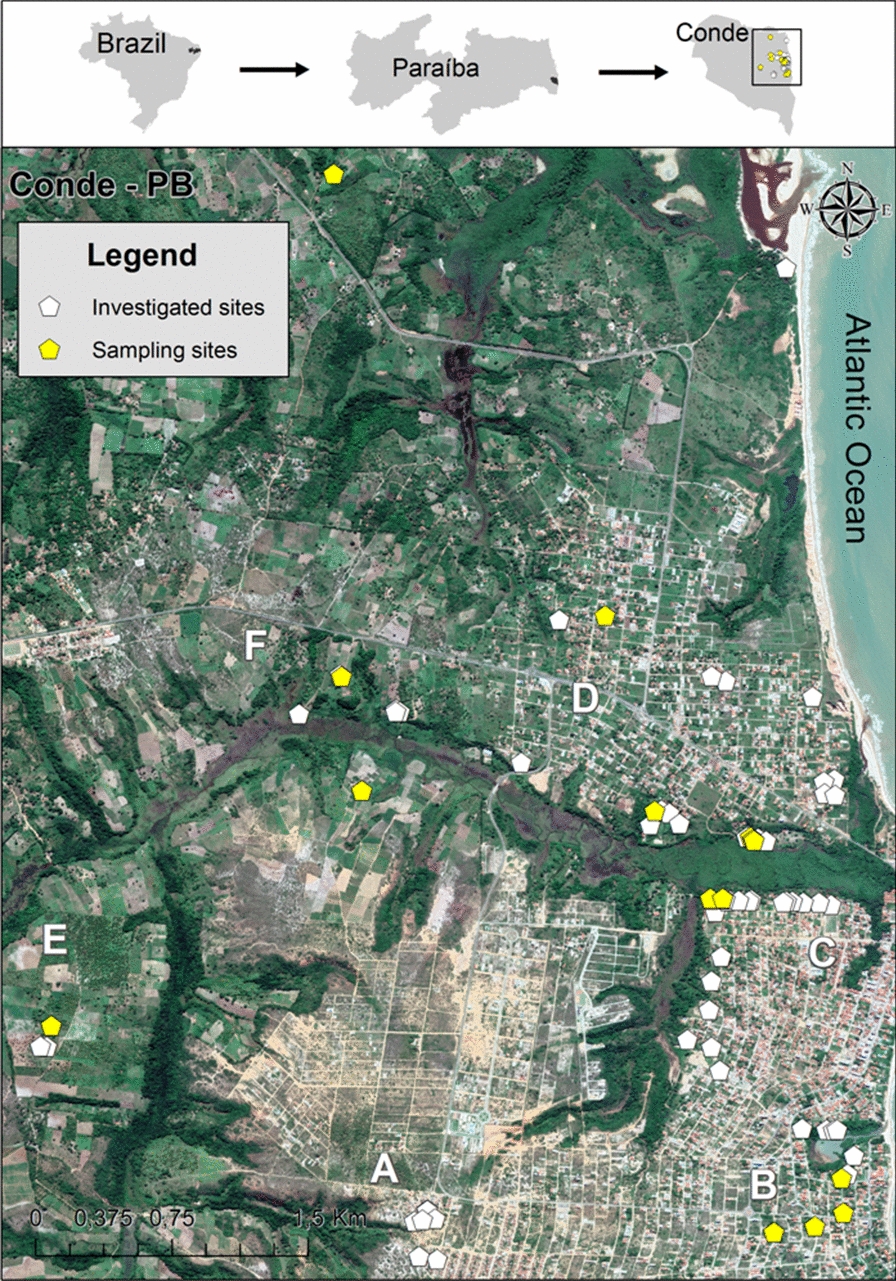
Location of the study area in Brazil, highlighting the state of Paraíba and the municipality of Conde (on top). The pentagons identify the distribution of 73 points where mosquito collections were performed, with white pentagons indicating investigated sites where *Anopheles* was not found and yellow pentagons indicating sampling sites or points of *Anopheles* collection. The uppercase letters* A–F* refer to the six main neighborhoods where mosquitoes were collected and are described in caption to Fig. 1

Adult mosquitoes of genus *Anopheles* were collected at different locations points in Conde (Fig. 2) between July and November 2019. The various capture methods employed included a battery-powered aspirator (Horst), mouth aspirator, the Shannon trap, BG-Sentinel traps, both with and without dry ice, and CDC-based light traps. Five monthly collections were carried out during the study period, with each collection conducted for 3 consecutive days in intradomicile, peridomicile and extradomicile areas. The collection points were determined using the following criteria: places of residence of cases, places of residence close to those of cases and natural breeding sites for mosquitoes in the locality.

The collection of mosquitoes using the battery-powered aspirator was carried out in the intra- and peridomicile environments during the period when *Anopheles* hematophagy was at its greatest peak (between 6 pm and 9 pm), with each aspiration lasting 20 min. The BG-Sentinel (with and without dry ice) and the CDC-based light traps were used in the peri- and extradomicile environments to capture mosquitoes during a period of approximately 12 h (6 pm to 6 am the next day). Mosquitoes were collected with a mouth aspirator between 6 pm and 8 pm from the walls in peridomicile areas and the surface of Shannon traps installed in the extradomicile environment. The sampling methods of mosquito collection were fixed for each “point of collection”. The variety of methods was used to increase the chances of catching mosquitoes, especially *Anopheles*, and to evaluate the best collection methods in this study area.

After each collection, the mosquitoes were immobilized with ether, and anophelines were separated from culicines based on external morphological characters [14]. Some mosquitoes were damaged during collections and sorting, especially those captured using BG-Sentinel and CDC-based light traps. Consequently, all anophelines were stored for species identification using molecular techniques (DNA Barcoding). These specimens were stored in individual tubes containing silica gel that were labeled with location, time and method of collection. The mosquitoes, properly identified and preserved, were sent to the Entomology Department laboratory of the Aggeu Magalhães Institute (IAM) for sexing and molecular identification of the species of anopheline. After sexing, each mosquito was sectioned, separating the head and chest (including the legs and wings) from the abdomen with the aid of sterile forceps and a scalpel.

### Molecular identification of species of* Anopheles*

DNA was extracted from the thoracic segment (including the legs and wings) of each mosquito individually, in accordance to the alcohol precipitation protocol [16]. After resuspension, DNA samples were quantified in Nanodrop 2000 (Thermo Fisher Scientific, Waltham, MA, USA) and then stored at − 20 °C for use in PCR analyses.

Conventional PCR was performed targeting the cytochrome* c* oxidase subunit 1 (COI) gene. For the amplification of this region, forward (5′-GGAGGRTTTGGAAAYTGAYTAGTYCC-3′) and reverse (5′-GCWGAWGTAAARTAAGCTCGWGTATC-3′) primers were used, generating an amplicon of approximately 698 bp [17]. PCR was performed using the GoTaq® Flexi DNA polymerase kit (Promega Corp., Madison, WI, USA), in accordance with the manufacturer’s instructions.

PCR products were sent for sequencing using the Applied Biosystems 3500 L genetic analyzer from IAM’s Technology Platforms Unit (NPT). All forward and reverse sequences from each individual were analyzed in the CodonCode Aligner Program (v. 3.7.1) for sequence quality assessment, editing, assembling of contigs and alignment. The nucleotide sequences produced from each sample were compared with the sequences deposited in databases such as Bold Systems (The Barcode of Life Data Systems; http://www.boldsystems.org) and GenBank, using nucleotide BLAST (BLASTn) to confirm the identity of the species collected.

### Spatial analysis

All mosquito collection sites, as well as all residences with cases of malaria registered in the municipality, were geo-referenced using a Vista Cx Garmin GPS (Global Positioning System) receiver (Garmin Ltd. Olathe, KS, USA), configured in the Universal Transverse Mercator (UTM) projection Datum SIRGAS 2000. The GPS data were then transferred and adjusted using GPS TrackMaker Pro software; the files (distribution of cases and points of capture of mosquitoes) were saved in Excel (Microsoft Corp., Redmond, WA, USA) spreadsheets to be used for spatial analysis of the data. Case data (number of cases per residence, cases of relapse and imported cases) and mosquitoes (presence of *Anopheles*, density and diversity per capture location) were incorporated into these spreadsheets, and thematic maps were created using the ArcGis 10 spatial data analysis software.

Kernel density estimation (KDE) was used to identify areas of greatest spatial risk for malaria transmission in Conde. The application of KDE made it possible to examine point distribution patterns (events) and to estimate the intensity of the process by location throughout the study region, thereby identifying areas of risk for occurrence of the event as “hotspots” [18]. KDE analyses were performed using the following parameters: the Jenks natural breaks classification method; adaptive bandwidth (the most suitable for analysis of local studies) [19, 20]; and unit area in square meters (m^2^). Municipal boundaries were used to determine the area marked out for analysis. The environmental background of the study area was examined using a satellite image from June 2019, with high 0.5-meter spatial resolution generated by means of panchromatic and multispectral sensors on board the Geoeye-1 satellite. This image is licensed under the Creative Commons Attribution 4.0 International License (available at http://creativecommons.org/licenses/by/4.0/legalcode) and DigitalGlobe, Inc. (http://www.digitalglobe.com/legal/website-terms-of-use).

## Results

In 2019, the municipality’s health system of Conde (BP) diagnosed 20 autochthonous cases and one imported case of malaria caused by *P. vivax*. The first case was reported on 3 March 2019, with symptoms reported to have started 1 week earlier (23 February 2019). The last case was reported on 22 October 2019. Among these 21 cases, three relapses were reported, including the imported case. In addition, two of the cases were registered at the same residence. The distribution of cases by gender was practically the same, with 11 cases in men and 10 cases in women. Age of the patients ranged from 14 to 64 years, with an average of 43.2 years (Table 1). The treatment regimen recommended by the Ministry of Health was adopted. This involves primary treatment of confirmed cases of *P. vivax* malaria with a combination of chloroquine and primaquine, for 3 and 7 days, respectively [8].

**Table 1:** Epidemiological data related to the autochthonous cases of malaria in Conde, Paraíba State, Brazil, 2019

Case number	Gender	Age (years)	Locality	Date of first symptoms (dd/mm/yyyy)	Date of diagnosis (dd/mm/yyyy)
1	F	35	Capadócia	23/03/2019	29/03/2019
2	M	53	Carapibus	28/03/2019	30/03/2019
3	F	40	Conjunto Rio do Ouro	04/04/2019	11/04/2019
4	M	64	Village Jacumã	21/04/2019	02/05/2019
5	F	26	Carapibus	26/05/2019	26/05/2019
6	F	27	Village Jacumã	14/05/2019	14/05/2019
7	M	25	Carapibus	29/05/2019	30/05/2019
8	F	52	Village Jacumã	27/05/2019	01/06/2019
9	M	57	Barauna	25/05/2019	04/06/2019
10	M	51	Conjunto Rio do Ouro	02/06/2019	04/06/2019
11^a^	M	44	Conjunto Rio do Ouro	27/05/2019	10/06/2019
12	M	32	Gurugi 2	10/06/2019	14/06/2019
13^a,b^	F	51	Village Jacumã	25/06/2019	25/06/2019
14	M	59	Village Jacumã	05/07/2019	12/07/2019
15	M	58	Village Jacumã	21/07/2019	26/07/2019
16	F	27	Capadócia	08/08/2019	12/08/2019
17	F	26	Capadócia	08/08/2019	12/08/2019
18*	F	29	Conjunto Rio do Ouro	25/08/2019	28/08/2019
19^a^	F	21	Capadócia	24/08/2019	28/08/2019
20^c^	M	14	Conjunto Rio do Ouro	29/08/2019	31/08/2019
21	M	23	Gurugi 2	07/10/2019	22/10/2019

dd, Day; F, female; M, male, mm, month; yyyy year

^a^Relapse (case 11: 23/08/2019; case 13: 27/08/2019; case 19: 07/02/2020)

^b^Imported case

^c^Two cases in the same residence

The cases were randomly spread, with the highest occurrence in the Vila de Jacumã neighborhood and the lowest in Baraúna (Table 1). Area A (see Fig. 1 and caption for designation of area), where the first case was registered, was also the area where one of the three relapses of the disease occurred (case 19). In the five cases recorded in area C, two were in the same household and there was one relapse (case 11). The largest number of cases were recorded in area D, which was also the area in which the imported case with relapse occurred (case 13). The smallest number of cases were in those areas furthest from the beach (E and F) (Fig. 1).

During the study period, 3713 mosquitoes were collected, 3390 of which were culicines and 323 anophelines. Nine species of anophelines were identified, the most abundant of which was *An. aquasalis* (38.9%), followed by *An. minor* (18.2%) and *An. albitarsis* (9.0%), and the least abundant were *An. braziliensis* and *An. sawyeri* (0.9%). All of the capture methods used collected at least one anopheline mosquito, but the most efficient were the CDC-based light trap, the BG-Sentinel with dry ice and the mouth aspirator, with these three capture methods collecting 87% of the anopheline mosquitoes (Table 2). Collection was performed at 73 points, and *Anopheles* mosquitoes were found at 16 of these (21.9%) (Fig. 2). As Table 2 shows, it was not possible to determine the species of around 12% of the *Anopheles* collected (*n* = 40), due to an insufficient quantity of DNA being available for analysis subsequent to extraction.

**Table 2:** Collection of *Anopheles* species according to capture methods in Conde, Paraíba State, Brazil, 2019

*Anopheles *species	Mosquito capture methods					Total
	CDC-based light trap	BG-Sentinel trap with dry ice	BG-Sentinel trap without dry ice	Castro insect capturer	Horst electric entomological aspirator	
*An. aquasalis*	83	35	2	1^b^	2	123
*An. minor*	1	7	–	36	15	59
*An. albitarsis*	4	7	–	4	14	29
*An. triannulatus*	22	4	–	2	–	28
*An. argyritarsis*	5	8	–	2	3	18
*An. peryassui*	–	–	–	13	2	15
*An. oswaldoi*	2	–	–	3	–	5
*An. braziliensis*	2	–	–	1	–	3
*An. sawyeri*	2	–	–	1	–	3
*Anopheles *spp.^a^	11	20	–	6	3	40
Total	132	81	2	69	39	323

**Fig. 3:**
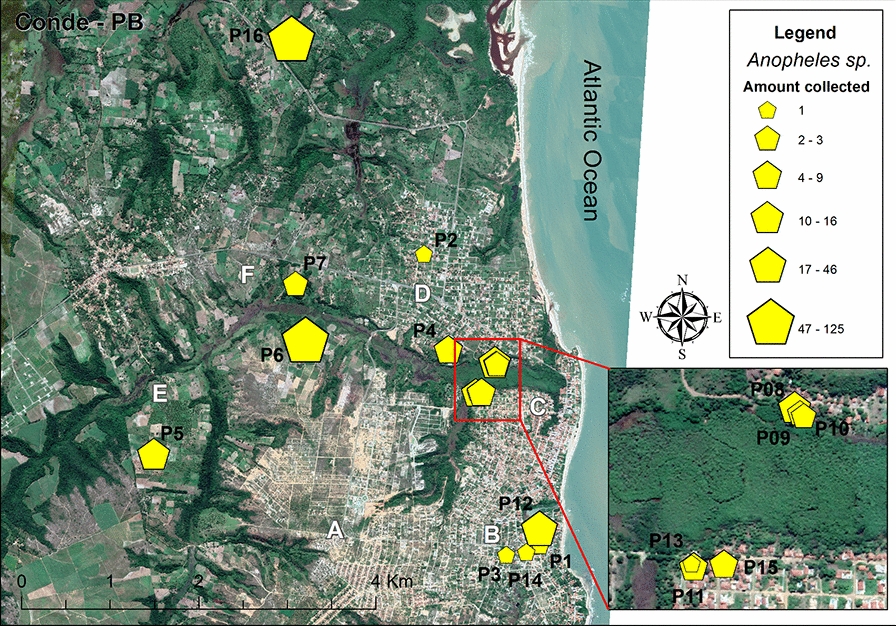
Map showing representation of *Anopheles* density at each of the 16 collection points (*P1*–*P16*, yellow pentagons). *Anopheles* density is represented by six pentagons that vary in size, ranging from 1 to 125 *Anopheles* collected at one point. The uppercase letters* A–F* refer to the six main neighborhoods where mosquitoes were collected and are described in caption to Fig. 1

**Fig. 4:**
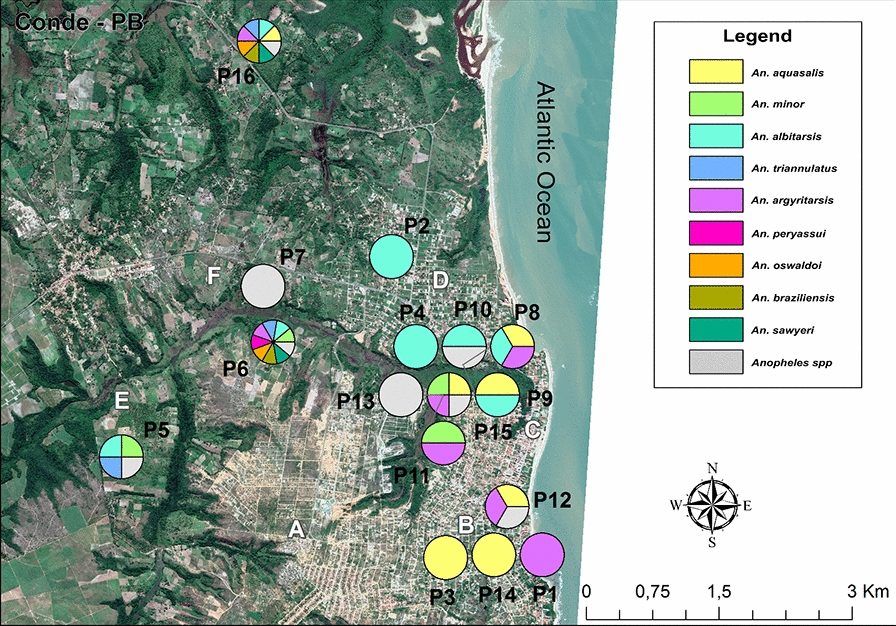
Map showing the diversity of *Anopheles* at each of the 16 collection points (*P1*–*P16*). Different *Anopheles* species are identified by colored circles, with different colors indicating different collection points. The uppercase letters* A–F* refer to the six main neighborhoods where mosquitoes were collected and are described in caption to Fig. 1

All sampling methods of mosquito collection were fixed and used in each location demarcated as “point of collection, except for mosquito collection using mouth aspirator on the Shannon trap that was performed only in point P6 and P8 (see Figs. 3, 4)

^a^It was impossible to determine the species due to the inadequatey amount of DNA for analysis after extraction

^b^Mosquito captured in Shannon trap

Figure 3 shows the spatial distribution of *Anopheles* collection points and the abundance of mosquitoes at each point. The greatest abundance of *Anopheles* specimens were collected at collection points P6 and P16, with these two points together accounting for 216 (66.9%) of all *Anopheles* specimens collected (Table 3). Figure 4 shows the diversity of mosquitoes at each capture point. It is noteworthy that the points with the greatest abundance are also those with the greatest diversity of species and that *An. aquasalis* and *An. albitarsis* were found at the largest number of collection points, as detailed in Table 3.

**Table 3:** Density of *Anopheles* species according to points of collection in Conde, Paraíba State, Brazil, 2019

Points of collection (P)	*An. aquasalis*	*An. minor*	*An. albitasis*	*An. triannulatus*	*An. argyritasis*	*An. peryassui*	*An. oswaldoi*	*An. braziliensis*	*An. sawyeri*	*Anopheles* spp.	Total
1	–	–	–	–	1	–	–	–	–	–	1
2	–	–	1	–	–	–	–	–	–	–	1
3	1	–	–	–	–	–	–	–	–	–	1
4	–	–	6	–	–	–	–	–	–	–	6
5	–	2	9	4	–	–	–	–	–	1	16
6	–	51	7	3	3	15	3	1	2	6	91
7	–	–	–	–	–	–	–	–	–	2	2
8	10	–	1	–	1	–	–	–	–	–	12
9	1	–	1	–	–	–	–	–	–	–	2
10	–	–	2	–	–	–	–	–	–	1	3
11	–	5	–	–	1	–	–	–	–	–	6
12	25	–	–	–	5	–	–	–	–	16	46
13	–	–	–	–	–	–	–	–	–	1	1
14	1	–	–	–	–	–	–	–	–	–	1
15	4	1	–	–	3	–	–	–	–	1	9
16	81	–	2	21	4	–	2	2	1	12	125
Total	123	59	29	28	18	15	5	3	3	40	323

All sampling methods of mosquito collection were fixed and used in each location demarcated as “point of collection, except for mosquito collection using mouth aspirator on the Shannon trap that was performed only in point P6 and P8 (see Figs. 3, 4)

**Fig. 5:**
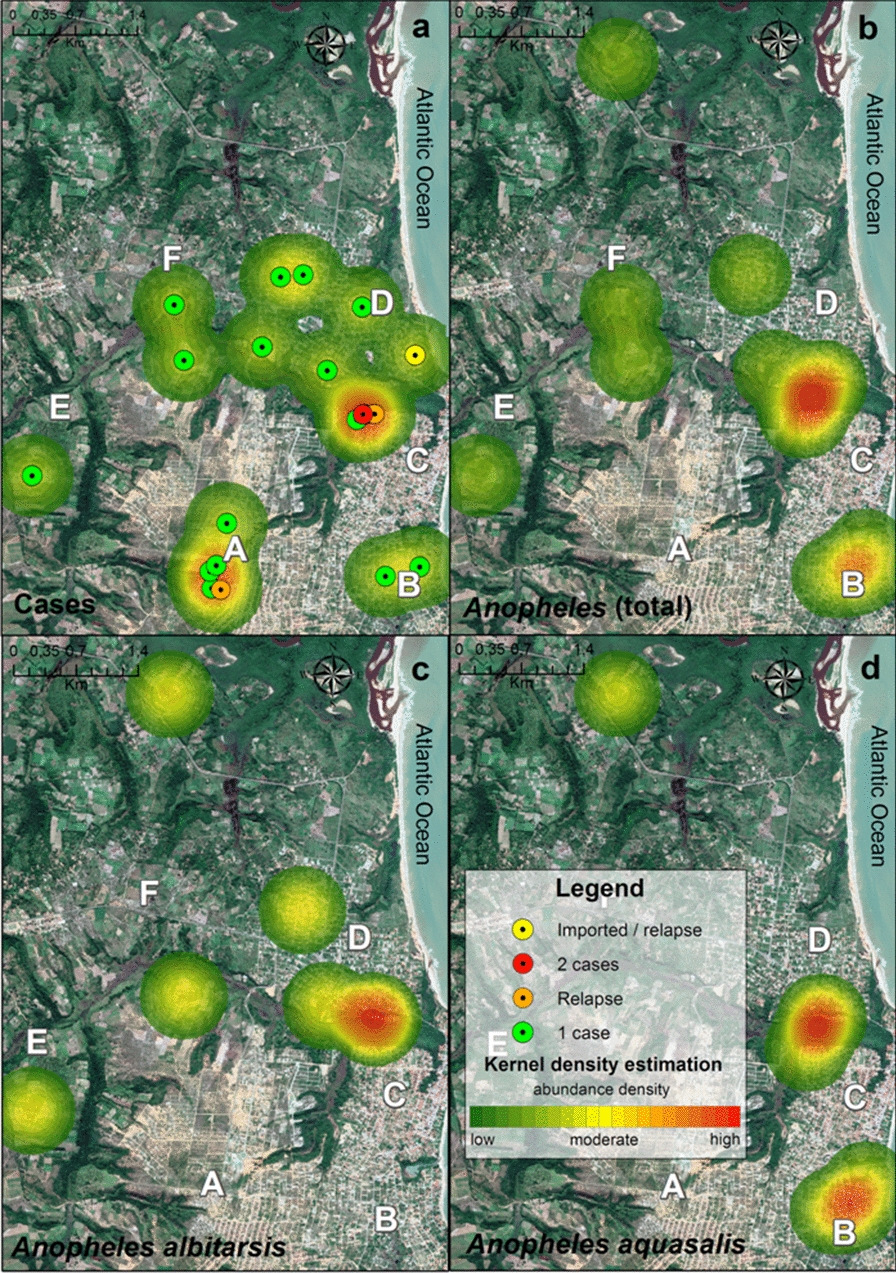
Maps showing the risk areas for occurrence of malaria and *Anopheles* as identified by kernel density estimation (KDE). Hotspots are identified ona color scale from green to red (low to high risk, respectively). **a** KDE of malaria cases based on distribution and number of cases by residences. The colored circles identify the distribution of malaria cases by geolocation, with each green circle indicating one case; the yellow circle indicating one imported case from Venezuela, with relapse after treatment; the 3 orange circles indicating three cases that showed relapse after treatment; and the two red circles indicating residences where two cases were registered **b** KDE of *Anopheles* collection points (total *Anopheles*). **c** KDE of *An. albitarsis* distribution by collecting points.** d** KDE of *An. aquasalis* distribution by collecting points. The uppercase letters* A–F* refer to the six main neighborhoods where mosquitoes were collected and are described in caption to Fig. 1

The areas with the greatest risk of occurrence of malaria based on analysis of the distribution of cases and mosquitoes collected are identified in Fig. 5. The highest concentration of cases was detected in area C, followed by area A. Areas B and D presented with a moderate risk for the occurrence of cases (Fig. 5a). Analysis of the distribution of total *Anopheles* by collection points (Fig. 5b), as well as the analysis of only the collection points of *An. albitarsis* and *An. aquasalis* (Fig. 5c, d), showed that area C was also the area that presented both the greatest risk of transmission and a higher risk for occurrence of both species. It is worth noting the existence of a second high-risk area for *An. aquasalis* in area B (Fig. 5d).

## Discussion

The occurrence of autochthonous cases of malaria in Conde is concerning for epidemiological surveillance services, since the location—a beach tourist region that receives visitors from national and international areas endemic for malaria—has all the features required for transmission and local maintenance of the disease [21]. Furthermore, the Brazilian Northeast has already experienced a major malaria epidemic, when *An. gambiae* was introduced into the region in the early 20th century [22]. The mosquito was later eradicated by action undertaken by the Northeast Malaria Service, created in 1939 in cooperation with the Rockefeller Foundation [23]. This past history provides clear evidence that the Northeast of Brazil has an environment conducive to the introduction of exotic mosquito species, as well as to the maintenance of the diverse range of *Anopheles* species already present in the region.

It is worth noting that Conde has a refugee program and voluntarily receives immigrants from Venezuela, a country with a high endemicity of malaria [1]. This may pose the risk of future introduction of new species of *Plasmodium*. Furthermore, the single imported case registered in the municipality during this outbreak of autochthonous cases did involve a Venezuelan immigrant recently arrived in the city. Upon arrival, this individual already exhibited symptoms of the disease and, as the epidemiological surveillance service was already effectively installed, diagnosis and treatment of the patient were immediate. However, this patient relapsed 2.5 months after the first diagnosis (Table 1). Such relapses are common when the cause of the infection is *P. vivax* since this species produces hypnozoites, which are dormant forms of the parasite that reside in the liver of the infected individual [8, 24]. There is therefore a risk of malaria epidemics occurring outside the Amazon region due to a large portion of the population no longer being immune to the pathogen [22].

With the exception of the last case of relapse, the other cases occurred within the expected period for relapse, generally within 3–9 weeks after treatment, which is common for most strains of *P. vivax* [8]. The last relapse occurred 5 months after the first diagnosis and, in this case, it may be that a new infection should be considered. However, it may also have been a relapse of the disease since *P. vivax* relapses have been described up to 24 weeks after treatment with chloroquine and primaquine [25].

Local spread is suspected to have occurred after the 2019 Carnival festivities, which took place during the first week of March. The hypothesis is that some infected tourists initiated the cycle of the parasite when *Anopheles* mosquitoes became infected through hematophagy. As the *P. vivax* cycle in the mosquito lasts about 12 days and the incubation period in humans lasts 13–17 days [8], this is roughly equal to the period for the onset of symptoms in the first case. The travelling habits of Brazilian population during periods of festivities and holidays may contribute to the occurrence of cases of malaria [26].

Most of the cases affected patients in early to mid-adulthood, even though malaria affects all age groups [27]. The age range can, however, vary according to regional characteristics and exposure [28], having a greater impact on the economically active population [29]. The occurrence of cases did not vary according to sex, even though a number of studies have pointed to a higher occurrence in men as a result of their greater exposure to high-risk situations [27–29]. This lack of sex-based variation in this region may be explained by the fact that many women also need to work in areas with a significant risk of transmission of the disease in order to support their families.

The cases occurred along an ecological corridor and always in the vicinity of mangroves and riparian forest alongside local rivers. This environment may be directly associated with the exophilic habits of the main potential vector species found in the locality—*An. aquasalis*,* An. albitarsis* and *An. argyritasis* [6]. The two cases occurring in the same residence, the imported case, and the relapses were all located precisely in those areas closest to this ecological corridor—a possible breeding site for mosquitoes.

Of the nine species of *Anopheles* identified in this study, seven were already registered in the State of Paraíba in the most recent survey of *Anopheles* [6]. *Anopheles oswaldoi* and *An. sawyeri* are reported here for the first time, and *An. noroestensis*,* An. kompi*,* An. intermediaius* and *An. bellator*, already reported in this state, were not found in the present study [6]. As can be seen in the results of this study related to the *Anopheles* investigation, the study site was thoroughly investigated, and area A was the only area in which *Anopheles* species were not found. This was the precise location at which the first case and one relapse were recorded. These occurrences may be associated with the area’s dryness and distance from possible breeding sites, both of which suggest that individuals with malaria in this area were infected in another location in the municipality. Transmission outside the home environment and that associated with the movement of human beings and work activities are a constant feature of the eco-epidemiology of malaria [26].

Of all the mosquito capture methods used in the study, the CDC-based light trap and the BG-Sentinel trap with dry ice performed best. While the high quality of the BG-Sentinel trap (with and without dry ice) has already been proven, the CDC-based light trap usually does not show good results when used for the collection of anopheline mosquitoes [30]. However, the latter proved to be extremely effective in the present study. Targeted collection using the mouth aspirator in peri- and extradomicile areas was the only method that collected all species found in the locality, thereby demonstrating both the efficiency of this method of capture and the exophilic behavior of these mosquitoes [14, 31]. Such behavior is reflected in the density and diversity of the species, showing that these species were found in greater abundance and diversity in the best-preserved areas located at some distance from urban agglomerations. This also indicates a low degree of anthropophilia [14].

*Anopheles aquasalis* is a species that reproduces preferentially in still and brackish pools of water (transient or semi-permanent, sunny or partially shaded) that occur in low-lying coastal areas, mainly in the Northeast of Brazil [14, 32]. It is zoophilic and exophilic, mostly preferring twilight. However, when it is present in large numbers in the absence of animals, the mosquito also uses human blood as a source of food. This is possibly what happened in Conde during this malaria outbreak, as the species also shows some degree of anthropophilia [14, 31]. This peculiarity has been observed in other anopheline mosquitoes, including mosquitoes of the *An. albitarsis* complex, which, for example, exhibit a peak of human blood-feeding in the early evening [14].

As shown from the risk maps presented here, the area that represents the highest risk for the occurrence of cases of malaria in Conde is precisely the area where the largest number of *Anopheles* capture points were recorded (area C). This is supported by the presence of the two main vector species of malaria in the extra-Amazon region—*An. albitarsis* and *An. aquasalis.* These findings suggest that these two species are the responsible vector for malaria transmission in this area. However, more information is needed to enhance understanding of the dynamics of malaria transmission in the municipality of Conde.

## Conclusions

The results of the present study clearly show that the Northeast Region of Brazil has an environment conducive to the maintenance of the malaria transmission cycle. There is a great diversity of *Anopheles* species in this region, especially *An. albitarsis* and *An. aquasalis*, capable of causing outbreaks or epidemics. In the current context of globalization, modernization of means of transport and large-scale movement of people, the city of Conde becomes a perfect setting for the occurrence and persistence of the disease.

Further studies of the material collected at this location will be needed to shed light on issues such as the role of each species of *Anopheles* in the transmission cycle through the diagnosis of vector infection by *P. vivax*. Further studies should also investigate the immune response of the local human population in order to ascertain whether malaria transmission is a recent event or whether cases of the disease have been underreported or reported as imported cases. These are decisive factors in the local health system’s efforts to control local transmission of malaria.

## Data Availability

All data and materials are available with the corresponding author.

## Abbreviations

DNA: Deoxyribonucleic acid
An.: *Anopheles*
COI: Cytochrome oxidase* c* subunit 1
FW: Forward
GPS: Global positioning system
IAM: Aggeu Magalhães Institute
KDE: Kernel density estimation
km^2^: Square kilometer
m^2^: Square meter
NPT: Technology platforms unit
P: *Plasmodium*
PB: Paraíba
PCR: Polymerase Chain Reaction
REV: Reverse
SINAN: Notifiable Diseases Information System
UTM: Universal Transverse Mercator
WHO: World Health Organization
